# Non-motor symptoms in motor neuron disease: prevalence, assessment and impact

**DOI:** 10.1093/braincomms/fcad336

**Published:** 2023-12-07

**Authors:** Emily Beswick, Deborah Forbes, Micheala Johnson, Judith Newton, Rachel Dakin, Stella Glasmcher, Sharon Abrahams, Alan Carson, Siddharthan Chandran, Suvankar Pal

**Affiliations:** Centre for Clinical Brain Sciences, the University of Edinburgh, Edinburgh, UK; Anne Rowling Regenerative Neurology Clinic, the University of Edinburgh, Edinburgh, UK; Euan MacDonald Centre for MND Research, the University of Edinburgh, Edinburgh, UK; Centre for Clinical Brain Sciences, the University of Edinburgh, Edinburgh, UK; Anne Rowling Regenerative Neurology Clinic, the University of Edinburgh, Edinburgh, UK; Euan MacDonald Centre for MND Research, the University of Edinburgh, Edinburgh, UK; Centre for Clinical Brain Sciences, the University of Edinburgh, Edinburgh, UK; Anne Rowling Regenerative Neurology Clinic, the University of Edinburgh, Edinburgh, UK; Euan MacDonald Centre for MND Research, the University of Edinburgh, Edinburgh, UK; Centre for Clinical Brain Sciences, the University of Edinburgh, Edinburgh, UK; Anne Rowling Regenerative Neurology Clinic, the University of Edinburgh, Edinburgh, UK; Euan MacDonald Centre for MND Research, the University of Edinburgh, Edinburgh, UK; Centre for Clinical Brain Sciences, the University of Edinburgh, Edinburgh, UK; Anne Rowling Regenerative Neurology Clinic, the University of Edinburgh, Edinburgh, UK; Euan MacDonald Centre for MND Research, the University of Edinburgh, Edinburgh, UK; Anne Rowling Regenerative Neurology Clinic, the University of Edinburgh, Edinburgh, UK; Euan MacDonald Centre for MND Research, the University of Edinburgh, Edinburgh, UK; Euan MacDonald Centre for MND Research, the University of Edinburgh, Edinburgh, UK; Human Cognitive Neurosciences, Psychology, School of Philosophy, Psychology and Language Sciences, the University of Edinburgh, Edinburgh, EH16 4SB, UK; Centre for Clinical Brain Sciences, the University of Edinburgh, Edinburgh, UK; Anne Rowling Regenerative Neurology Clinic, the University of Edinburgh, Edinburgh, UK; Centre for Clinical Brain Sciences, the University of Edinburgh, Edinburgh, UK; Anne Rowling Regenerative Neurology Clinic, the University of Edinburgh, Edinburgh, UK; Euan MacDonald Centre for MND Research, the University of Edinburgh, Edinburgh, UK; UK Dementia Research Institute, the University of Edinburgh, Edinburgh, EH16 4SB, UK; Centre for Clinical Brain Sciences, the University of Edinburgh, Edinburgh, UK; Anne Rowling Regenerative Neurology Clinic, the University of Edinburgh, Edinburgh, UK; Euan MacDonald Centre for MND Research, the University of Edinburgh, Edinburgh, UK

**Keywords:** motor neuron disease, non-motor symptoms, prevalence, impact, survey

## Abstract

People with motor neuron disease often experience non-motor symptoms that may occur secondary to, or distinct from, motor degeneration and that may significantly reduce quality of life, despite being under-recognized and evaluated in clinical practice. Non-motor symptoms explored in this population-based study include pain, fatigue, gastrointestinal issues, poor sleep, low mood, anxiety, problematic saliva, apathy, emotional lability, cognitive complaints and sexual dysfunction. People registered on the Clinical Audit Research and Evaluation of motor neuron disease platform, the Scottish Motor Neuron Disease Register, were invited to complete a questionnaire on non-motor symptoms and a self-reported Amyotrophic Lateral Sclerosis Functional Rating Scale. The questionnaire comprised a pre-defined list of 11 potential non-motor symptoms, with the opportunity to list additional symptoms. A total of 120 individuals participated in this cross-sectional study, a 39% response rate of those sent questionnaires (*n* = 311); 99% of participants recruited (*n* = 120) experienced at least one non-motor symptom, with 72% (*n* = 120) reporting five or more. The symptoms most often reported were pain and fatigue (reported by 76% of participants, respectively). The symptoms reported to be most impactful were gastrointestinal issues (reported as ‘severe’ by 54% of participants who experienced them), followed by pain and problematic saliva (51%, respectively). Lower Amyotrophic Lateral Sclerosis Functional Rating Scale scores, indicating more advanced disease and being a long survivor [diagnosed over 8 years ago; Black *et al*. (Genetic epidemiology of motor neuron disease-associated variants in the Scottish population. *Neurobiol Aging*. 2017;51:178.e11-178.e20.)], were significantly associated with reporting more symptoms; 73% of respondents were satisfied with the frequency that non-motor symptoms were discussed in clinical care; 80% of participants indicated they believe evaluation of non-motor symptom is important to include as outcomes in trials, independent of their personal experience of these symptoms. The preferred method of assessment was completing questionnaires, at home. The overwhelming majority of people with motor neuron disease report non-motor symptoms and these frequently co-occur. Pain, fatigue, gastrointestinal issues, sleep, mood, anxiety, problematic saliva, apathy, emotional lability, cognitive complaints and sexual dysfunction are prevalent. People with motor neuron disease who had worse physical function and those who were long survivors were more likely to report more symptoms. Where reported, these symptoms are frequent, impactful and a priority for people with motor neuron disease in clinical care and trial design.

## Teaser

Beswick *et al.* report non-motor symptoms (pain, fatigue, gastrointestinal, sleep, mood, anxiety, problematic saliva, apathy, emotional lability, cognitive complaints and sexual dysfunction) are prevalent in people with motor neuron disease and frequently co-occur. These are identified as priority by people with motor neuron disease in clinical care and trial design.

## Graphical Abstract

**Graphical Abstract:**
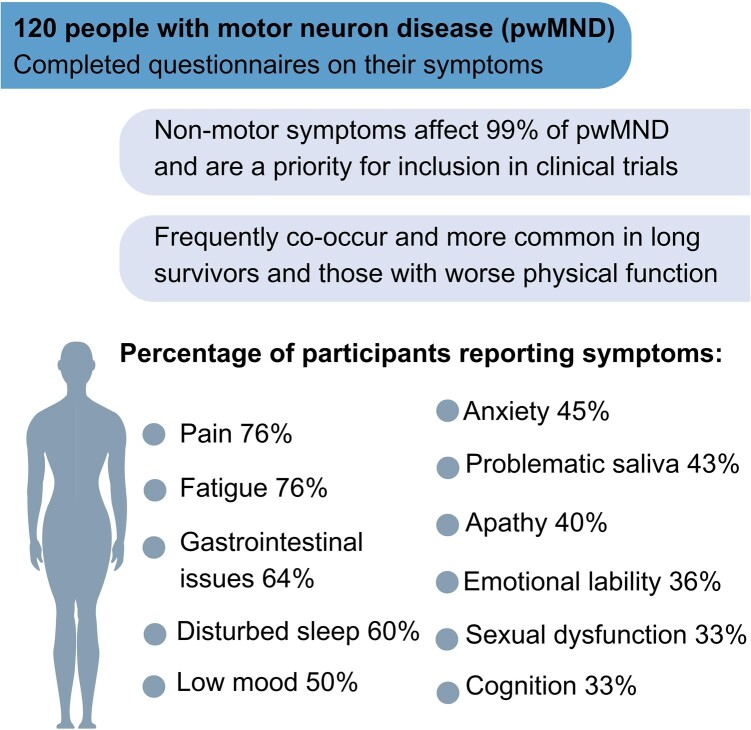

## Introduction

### Non-motor symptoms

Non-motor symptoms such as low mood, anxiety, cognitive complaints, behavioural change, fatigue, pain, disturbed sleep, problematic saliva, sexual dysfunction and gastrointestinal issues are increasingly acknowledged as prevalent and impactful features of motor neuron disease.^1-3^ Although motor neuron disease is increasingly recognized as a multi-system disorder, these symptoms remain under-explored, with only 1% of motor neuron disease-related publications focusing on non-motor symptoms.^4^ The prevalence of these symptoms has previously been evaluated using adapted assessment tools validated for other neurological conditions^5^ and symptom-specific studies.^6^ The types of non-motor symptoms evaluated vary across studies,^5,7^ with limited exploration of participant-reported feedback from people with motor neuron disease on the importance and impact of these symptoms.^3^

Non-motor symptoms can occur due to motor neuron disease pathology, and as a result of motor degeneration, symptoms may be associated with pathology affecting different neuroanatomical regions,^5^ broadening understanding of the aetiopathogenesis of motor neuron disease and providing insights into wider neuroanatomical dysfunction.^8^ Symptoms such as cognitive and behavioural change, manifestation due to the pathology of the disease, which includes frontotemporal cerebral dysfunction.^9^ Inefficient saliva clearance from bulbar motor dysfunction, pain and gastrointestinal issues from inability to move regularly and disturbed sleep due to pain or anxiety, may considered as results of the impact of motor decline. Additional symptoms, such as low mood, anxiety, fatigue and sexual dysfunction, may be more complex aetiologically, occurring directly as a result of motor neuron disease pathology for some people, but result of receiving a difficult diagnosis and experiencing progressive disability for others.

Motor neuron disease is a multi-system disorder with non-motor symptoms that are poorly understood, and their impact upon the individual is not yet well established. An overview of the symptoms, which will be explored in this study, and their reported prevalence from prior research, is provided in Table 1. The prevalence and progression of these symptoms have also been shown to vary depending on disease characteristics^16^ and increase in frequency as the disease progresses^5^; we will investigate the associations between clinical and demographic features with the number of non-motor symptoms participants report.

**Table 1:** Non-motor symptoms explored in this study

Symptom	Prevalence	Features of symptom
Pain	50–85%^10^	Pain became more problematic over time and reduced quality of life.^10^ May be responsive physical therapy^11^ and pharmacological intervention.^12^
Sleep	Sleep disturbance and poor quality sleep are frequently reported but exact prevalence depends on the specific sleep-related issue^49^	Sleep quality, length of sleep and rapid eye movement duration can be improved in people with motor neuron disease utilizing non-invasive ventilation.^13^
Fatigue	Up to 44% of person with motor neuron disease	Co-occurs with depression in 15% of individuals.^14^ Sleepiness is a key aspect of fatigue in people motor neuron disease, along with reduced alertness, loss of stamina and lack of energy.^7^
Neuropsychiatric	19.7%, 70% of which were mood disorders and 31.67% neurotic disorders (inclusive of anxiety, stress-related and somatoform disorders)^15^	Elevated in comparison with rates in the general population, 6.9% of whom fulfil diagnostic thresholds for major depressive disorder and 14% an anxiety disorder.^50,51^
Cognitive complaints	Experienced by 30–50% of person with motor neuron disease	Detrimental to an individual’s quality of life and prognosis.^52,53^
Behaviour change	28% indicated abnormally high levels of apathy^54^ 50% experience emotional lability^54^	Apathy is the most commonly reported behavioural change, measured with the Dimensional Apathy Scale.^55^ Emotional lability is characterized by some changes in emotionality and emotion regulation.^54^
Problematic saliva	37.5% of people with motor neuron disease^56^	Problematic saliva production and handling in motor neuron disease is characterized by thickened saliva and sialorrhoea. Detrimental to an individual’s well-being, increase the risk of respiratory complications and exacerbate dysarthria.^57^
Gastrointestinal	16–83%, depending on the specific symptom^7^	Symptoms such as constipation, nausea, vomiting, acid reflux or excessive flatulence.^58^ May occur as a response to interventions (particularly new drug treatments or non-invasive ventilation), secondary to increasingly physical disability or as indicative of wider nervous system degeneration.^7,59^
Sexual dysfunction	Interest in sex reported to decrease 28% after an motor neuron disease diagnosis^60^	Not directly affected by motor neuron disease but the impact of an motor neuron disease diagnosis on sexuality, intimacy and libido remain relevant to exploring the a more holistic impact of non-motor symptoms of the condition on the individual^61^ Changes in sexual behaviour can have on an individual’s quality of life^61,62^; sexuality is often not discussed in routine clinical consultations or included in research design.

This study explores how frequently people with motor neuron disease report non-motor symptoms, co-occurrence across symptoms and respondents’ perception of the clinical care they receive regarding these issues. The impact of the presence of these symptoms on the individual was also evaluated, with symptoms defined as impactful if they were reported as significant to respondents, a definition encompassing both the perceived severity and burden of the symptom on the individual. It also considers how important people with motor neuron disease report it is to address these non-motor symptoms in clinical trial design, whether this is to explore potential additional therapeutic benefit of candidate drugs or to provide a complete picture of potential side-effects.

### Assessment in clinical care

In a condition such as motor neuron disease, where no cure is currently available, the primary focus of clinical care is to provide symptomatic relief and improve an individual’s quality of life.^17^ The broad range of non-motor symptoms previously reported,^18^ and their significant effect on quality of life,^17^ has highlighted the need for further research into the presence and impact of a broad range of non-motor symptoms in motor neuron disease.

In the UKL, evidence-based management of people affected by motor neuron disease is supported by the National Institute of Clinical Excellence 2016 guidelines.^19^ Approaches for some non-motor symptoms’ diagnosis and management are included in these guidelines, but a clear focus on the motor features of respiratory failure, muscular weakness and bulbar symptoms remains. Key challenges for the identification of non-motor symptoms in clinical practice are the emerging awareness of these symptoms’ association with motor neuron disease, from both the clinician and patient perspective, and the lack of validated disease-specific screening tools available.^4^ This is further confounded by the inconsistency in published literature relating to which non-motor symptoms are associated with motor neuron disease.^20^

The Trajectories of Outcomes in Neurological Conditions study is multi-phase exploration of quality of life determinants in motor neuron disease; the Trajectories of Outcomes in Neurological Conditions study reported the prevalence of pain, depression and anxiety and the resulting negative impact on an individual’s quality of life.^21^ Despite evidence from the Trajectories of Outcomes in Neurological Conditions group that people with motor neuron disease and their clinical team believe that several of these symptoms can affect the quality of life,^22^ non-motor symptoms are often under-reported by patients in clinical consultations more generally, perhaps due to a lack of awareness that they are related to motor neuron disease progression.^23^

The National Institute of Clinical Excellence clinical guidance^19^ directly addresses cognitive and behavioural change as symptoms of motor neuron disease that may precede motor dysfunction and as aspects of functioning that need to be continually monitored. Guidelines for managing the psychological and social impact of motor neuron disease, changes relating to sexual symptoms and intimacy and problematic saliva are also provided. Problematic sleep and fatigue are considered as secondary effects of respiratory dysfunction. Guidelines on symptom-focused treatment^24^ encourage a broader conceptualization of motor neuron disease as a multi-system disorder with non-motor symptoms as areas that also require effective management and treatment.

### Evaluation of non-motor symptoms in clinical trials

Guidelines on designing and running trials for people with motor neuron disease have also encouraged the exploration of non-motor symptoms as additional outcome measures. The Airlie House recommendations on trial design highlight the potential for cognitive or behavioural assessments to be included as primary or secondary outcome measures.^25^ The European Medicines Agency ALS Trial Guidelines also indicate the importance of also evaluating mood.^26^

Including assessment of non-motor symptoms as additional outcome measures in clinical trials enables researchers to provide a more complete picture of the potential side-effects and additional benefits of prospective disease-modifying treatments. Expanding our concept of treatment when evaluating a candidate drug, to include the potential for beneficial impact on non-motor symptoms, may identify drugs that have a significant impact on the quality of life and disability experienced by people with motor neuron disease.^27,28^ Despite this guidance, we have recently reported that non-motor symptoms have been insufficiently addressed in trial design,^29,30^ and where these symptoms were evaluated, it was often with assessments that were not designed to evaluate non-motor symptoms in people with a progressive physical decline or communication impairment.^29,30^

### Aims

The aims of this study were (i) to explore how frequently people with motor neuron disease report symptoms of low mood, anxiety, cognitive complaints, behavioural change, fatigue, pain, disturbed sleep, problematic saliva, sexual dysfunction and gastrointestinal issues occurring and the clinical characteristics associated with a number of symptoms reported; in addition, (ii) to investigate how impactful these symptoms are for people with motor neuron disease and how many individuals reporting a symptom report it to be significant to them; also (iii) to explore how frequently people with motor neuron disease report that non-motor symptoms are assessed in clinical care; and (iv) to understand if people with motor neuron disease feel these symptoms are important to consider in clinical trial design.

### Objectives

The objective of this study was to establish the frequency and impact of non-motor symptoms by inviting people with motor neuron disease to complete questionnaires on how their disease affects them. These questionnaires were designed specifically for this study with input from people with motor neuron disease and their clinical team. Data from these questionnaires will be combined with clinical data from participants’ records on the Scottish Motor Neuron Disease register.

### Hypotheses

We hypothesize that non-motor symptoms will be frequently reported by people with motor neuron disease, often co-occur and be more common in individuals with worse physical function and older in age. In addition, we hypothesize that people with motor neuron disease may not find the frequency that these non-motor symptoms are assessed in clinical care, or evaluated in trial design, to be sufficient. As a result, we hypothesize that people with motor neuron disease may be supportive of including non-motor symptom assessment in future clinical trial design.

## Materials and methods

### Participant recruitment

The Scottish Motor Neuron Disease Register [(Clinical Audit Research and Evaluation (CARE) of motor neuron disease] has an established record of 99% case ascertainment of individuals living with motor neuron disease in Scotland. CARE of motor neuron disease provides longitudinal clinical phenotyping, including many of the clinical and demographic variables used in this study to supplement participant questionnaires. CARE of motor neuron disease is also a register of people with motor neuron disease who are interested in additional research participation and was used to facilitate recruitment to the current study, to minimize the risk of bias in all individuals who provided consent to be contacted about research invited to participate in the current study.^31^ No additional exclusion or inclusion criteria were applied.

### Patient consent

All participants in this study provided written consent alongside their questionnaires; the consent was obtained according to the Declaration of Helsinki. Ethical approval was provided for this study on 19 October 2021 (Yorkshire and The Humber, South Yorkshire Research Ethics Committee: 21/YH/0226).

### Data collection

Source data from participant-completed questionnaires were collected using paper questionnaires or an online survey, depending upon participant preference, to ensure participants with physical disability, speech impairment or inexperience with technology were not alienated from participating.

Within the questionnaire pack was the Amyotrophic Lateral Sclerosis Functional Rating Scale—Revised (ALS-FRS(R)), a questionnaire-based assessment, evaluating the presence and resulting disability, of physical symptoms commonly affecting people with motor neuron disease.^32^ The participant-reported version of the ALS-FRS(R) demonstrated high inter-rater and intra-rater reproducibility^33^ and was shown to be suitable for remote digital assessment.^34^ In addition to data collection through questionnaires, this project involved a data request to CARE of motor neuron disease for clinical information on participants.

### Non-motor symptom selection

The pre-specified list of 11 symptoms explored in this study was selected based on current literature, with input from motor neuron disease clinical specialists, people with motor neuron disease and their caregivers. An opportunity to highlight any symptoms that were impactful to each individual, but not included in the pre-specified list, was also provided in the questionnaire. The 11 aspects of health explored were pain, fatigue, gastrointestinal issues, poor sleep, low mood, anxiety, problematic saliva, apathy, emotional lability, cognitive complaints and sexual dysfunction. Table 1 reports data from previous literature, where available, on the prevalence and features of these symptoms in people with motor neuron disease.

In addition, we asked participants their preference on how these symptoms were assessed. Participants were asked to indicate if they found five types of assessment methods ‘acceptable’, or if they would ‘prefer not to’, ‘not sure’ was also available to offer a neutral response option too. These response options were assigned a numerical code, ‘acceptable’ = 3, ‘not sure = 2’, and ‘prefer not to’ = 1, or in order for responses to be ranked by preference.

### Study questionnaire

The questionnaire was developed specifically for this study, and a full copy is provided in the appendices. The questionnaire content was designed with input from members of the multi-disciplinary care team for people with motor neuron disease, expert neurologists and a motor neuron disease nurse consultant. In addition, people with motor neuron disease attending routine National Health Service clinics provided feedback on the early design. The selection of pre-specified potential non-motor symptoms was based on previous literature.^2,3^ People with motor neuron disease, and their caregivers, provided feedback on the questionnaires in the final stages to ensure the symptom list was comprehensive.

### Statistical analysis plan

Descriptive statistics were used to explore the characteristics of the sample, considering demographic, phenotypic and clinical data followed by ascertainment of the total number of non-motor symptoms reported (defined as a ‘yes’ response to the list of 11 pre-specified symptoms) are associated with age at participation, age at diagnosis, years since diagnosis and ALS-FRS(R) score using a series of linear regression models. Analysis of variance and *t*-tests were used to explore the same association of the total number of non-motor symptoms with categorical variables: long survivorship (defined as greater than 7 years since diagnosis), use of interventions, gender and disease sub-type. Missing data will be represented as not available values and the variable excluded from the analysis of that variable; respondents with over half of their data points missing will be excluded from the analysis.

A binary logistic regression was used to explore if the number of non-motor symptoms an individual reported is associated with their response to the questionnaire item ‘Is it important to you that trials for new drug treatments also consider symptoms like these [non-motor symptoms] as well?’.

Preference for the assessment method was represented using descriptive statistics, to consider the number of responses to each preference level for the options of assessment.

## Results

### Sample characteristics

A total of 120 people with motor neuron disease completed the questionnaires between 13 November 2021 and 23 February 2022, 109 in paper format and 11 online. This sample represents 23% of the 532 people living with motor neuron disease in Scotland during the recruitment period and 39% of the group (*n* = 311) who provided consent to be approached for recruitment to research studies such as this.

Despite a potential for responder bias, the sample characteristics appear to be generally representative of the wider motor neuron disease population^35^; Table 2 provides an overview of the demographic and clinical characteristics of study participants. The gender divide of participants and the frequency of bulbar symptom onset were akin to previous registry studies in the Scottish^36^ and American motor neuron disease populations.^37^ The use of riluzole,^36^ non-invasive ventilation^38^ and gastrostomy insertion^13^ in study participants was also representative of the wider motor neuron disease population in Europe.

**Table 2:** Characteristics of non-motor symptoms in motor neuron disease participants

Characteristics	Overall (%) (*N* = 120)
Age at diagnosis, mean (SD) (years)	60 (12)
Age at participation, mean (SD) (years)	65 (11)
Survival length, mean (SD) (years)	5 (6)
*Long survivor > 8 years (%)*	23 (19)
Males, no. (%)	76 (63)
Motor neuron disease sub-type, no. (%)	
*Amyotrophic lateral sclerosis*	70 (58)
*Progressive lateral sclerosis*	21 (18)
*Progressive bulbar palsy*	11 (9)
*Progressive muscular atrophy*	5 (4)
*SOD1 amyotrophic lateral sclerosis*	2 (1.5)
*Motor neuron disease–frontotemporal dementia*	2 (1.5)
*No data*	9 (8)
Bulbar onset (%)	21 (18)
Current intervention use (%)	
*Riluzole*	44 (37)
*Non-invasive ventilation*	26 (22)
*Gastrostomy*	22 (18)
Referral to speech and language therapy	73 (61)
ALSFRS(R)	
*Mean*	30
*Range*	3–47
*SD*	9

Age at diagnosis of participants was lower than Scottish^36^ and English^14^; motor neuron disease registries have previously indicated, which may be explained by the greater prevalence of people with the progressive lateral sclerosis sub-type in this cohort, as progressive lateral sclerosis often presents at a younger age and is slower to progress than the amyotrophic lateral sclerosis sub-type.^39^ The greater representation of long survivors in the study cohort, defined as individuals with survival from diagnosis greater than 8 years,^1^ than the wider motor neuron disease population may also partially explain the younger age of study participants as long survivorship is associated with younger age at onset.^40^

### Total number of non-motor symptoms reported

#### Presence, frequency and impact

Ninety-nine per cent of participants experienced at least one non-motor symptom, with 72% reporting five or more non-motor symptoms. The presence, impact and reported frequency of these symptoms are shown in Table 3.

**Table 3:** Frequency, severity and impact of non-motor symptoms

Symptom	Frequency of reporting as ‘present’ *n* (%)	Frequency of reporting as ‘significant’ *n* (%)	Frequency of symptom occurrence in previous fortnight *n* (%)
Pain	91 (76)	27 (51)	*Every day*, 34 (40) *Most days*, 30 (35) *A few days*, 21 (25)
Fatigue	91 (76)	45 (49)	*Every day*, 32 (36) *Most days*, 24 (27) *A few days*, 32 (36)
Gastrointestinal	77 (64)	42 (54)	*Every day*, 14 (19) *Most days*, 24 (33) *A few days*, 35 (48)
Sleep	72 (60)	28 (39)	*Every day*, 27 (39) *Most days*, 22 (32) *A few days*, 20 (29)
Low mood	60 (50)	45 (48)	*Every day*, 7 (13) *Most days*, 7 (13) *A few days*, 42 (75)
Anxiety	54 (45)	13 (24)	*Every day*, 8 (16) *Most days*, 10 (20) *A few days*, 32 (64)
Saliva	52 (43)	27 (51)	*Every day*, 27 (56) *Most days*, 11 (23) *A few days*, 10 (21)
Apathy	48 (40)	21 (41)	*Every day*, 7 (15) *Most days*, 17 (35) *A few days*, 23 (47)
Emotional lability	43 (36)	15 (33)	*Every day*, 1 (3) *Most days*, 13 (33) *A few days*, 26 (65)
Cognition	40 (33)	18 (41)	*Every day*, 7 (18) *Most days*, 14 (41) *A few days*, 18 (46)
Sexual Dysfunction	40 (33)	20 (43)	*Every day*, 22 (61) *Most days*, 8 (22) *A few days*, 6 (17)

The most frequently reported non-motor symptoms were fatigue and pain, each affecting 76% of participants; 40% of participants who reported that they experienced pain described that their pain occurred ‘every day’ in the previous fortnight.

To ensure a more complete picture of the symptoms, experienced participants were also asked to report any additional symptoms that were impactful to them, including any non-motor symptoms not included in the pre-specified list and motor symptoms. A summary of the most frequently reported symptoms is presented in Table 4, with the full data available in Supplementary Table 1, as this includes the frequency with which these symptoms were reported.

**Table 4:** Participant-reported impactful symptoms

Symptom grouping	Symptom reported	Number of times symptom is identified as present by participants^a^	Frequency of symptom reported as occurring in the past 2 weeks^b^	
			Over half the days (%)	Under half the days (%)
Muscle	*Weakness/stiffness*	56	55 (98)	1 (2)
	*Fasciculations*	8	5 (63)	2 (3)
Mobility	*Loss of limb function*	45	42 (93)	1 (2)
	*Walking*	36	35 (97)	-
Oral	*Speech*	34	30 (88)	2 (6)
	*Swallow/choking*	24	22 (92)	2 (8)
Tiredness	*Fatigue*	33	31 (94)	2 (6)
	*Sleep*	14	13 (93)	1 (7)
Pain	*Pain*	26	26 (100)	-
	*Cramps*	19	13 (68)	6 (32)
Quality of life	*Independence in activities of daily living*	17	17 (100)	-
	*Quality of life*	4	2 (50)	2 (50)
Cognition or behaviour	*Behaviour change*	9	4 (45)	5 (55)
	*Emotional lability*	7	3 (43)	4 (57)
Gastrointestinal	*Constipation*	9	4 (45)	5 (55)
	*Gastrointestinal (unspecified)*	3	2 (67)	1 (33)
Mood	*Low mood*	7	4 (57)	3 (43)
	*Anxiety*	3	2 (67)	1 (33)
Continence	*Incontinence*	2	2 (100)	-
	*Nocturia*	1	-	-

aNote that due to missing data patterns, frequency of identification may be greater than frequency of occurrence or impact and percentages may not total 100%. bRepresented as a percentage of the number of people who reported this symptom as occurring to them.

#### Participant characteristics and non-motor symptoms

There was a statistically significant relationship between ALS-FRS(R) score and the total number of non-motor symptoms reported (*R*^2^ = 0.056, *F*(1, 111) = 6.60, *P* < 0.0010; Table 5). As the ALS-FRS(R) score decreases (indicative of worsening physical function), the number of non-motor symptoms reported increased significantly (*β* = −0.88, *P* = 0.01).

**Table 5:** Linear regressions to explore ALSFRS(R), years since diagnosis and age with total number of non-motor symptoms reported

	*B*	95% confidence interval	*β*	*r*(112)	*r* ^2^	*P*-value
Intercept *ALSFRS(R)(score)*	35.75** −0.79*	(32.05–39.44) (−1.40, −0.18)	−0.24	−0.24*	0.052	<2e^−16^*** 0.0122
Intercept * Age at participation*	4.81** 0.01	(2.20–7.41) (−0.03, 0.05)	0.06	0.06	0.003	0.00039 0.544
Intercept * Age at Diagnosis*	4.84** 0.01	(2.48–7.19) (−0.03, 0.05)	0.06	0.06	0.004	8.78e^−05^*** 0.519
Intercept *Years Since Diagnosis*	5.50** 0.02	(4.94–6.07) (−0.06, 0.10)	0.05	0.05	0.002	<2e^−16^*** 0.622

*Indicates significance at *P* < 0.05. **Indicates significance at *P* < 0.01. ***Indicates significance at *P* < 0.00.

The number of reported non-motor symptoms did not significantly increase as age at diagnosis (*R*^2^ = 0.004, *F*(1, 111) = 0.420, *P* = 0.52) or age at participation increased (*R*^2^ = 0.003, *F*(1, 111) = 0.3697, *P* = 0.54). The number of non-motor symptoms reported was not significantly associated with years since diagnosis (*R*^2^ = 0.002, *F*(1, 111) = 0.273, *P* = 0.62). However, there was a significant difference in the number of non-motor symptoms reported between long survivors (*M* = 6.8, SD = 2.3) and others (*M* = 5.3, SD = 2.3), *t*(119) = −2.9, *P* = 0.005, with long survivors (*n* = 23) reporting more non-motor symptoms. The effect size, as measured by Cohen’s *d*, was *d* = −0.67, indicating a medium effect.

There was no significant difference in number of non-motor symptoms reported between men (*M* = 5.6, SD = 2.4) and women (*M* = 5.4, SD = 2.2), *t*(119) = −0.48, *P* = 0.63, or between the two largest disease sub-types in the sample, amyotrophic lateral sclerosis (*n* = 67) and progressive lateral sclerosis (*n* = 21) (*F*(1, 87) = 0.003, *P* = 0.96). Rarer sub-types under-represented in this study’s sample (including motor neuron disease amyotrophic lateral sclerosis, SOD1 amyotrophic lateral sclerosis, progressive bulbar palsy, progressive muscular atrophy and those with no data on sub-type) were removed from this section of the analysis to improve power to detect an effect.

We explored the influence of riluzole, non-invasive ventilation and feeding tube use (never taken, currently taking and discontinued) on the prevalence of non-motor symptoms. The use of riluzole (*F*(3, 117) = 0.22, *P* = 0.91), non-invasive ventilation (*F*(3, 117) = 0.62, *P* = 0.54) or a feeding tube (*F*(3, 117) = 0.35, *P* = 0.70) was not associated with the number of non-motor symptoms an individual reported (Table 6).

**Table 6:** Analysis of variance to explore intervention use and disease sub-type with total number of non-motor symptoms reported

	Sum of squares	DF	Mean square	*F*(88)	*η* ^2^	*P*-value
*Disease sub-type* ^a^	0.0	1	0.014	0.003	2.90	0.96

	Sum of squares	DF	Mean square	*F*(110)	*η* ^2^	*P*-value
	29.3	5	5.85	1.04	0.05	0.40
*Riluzole use*	1.0	2	0.48	0.22	0.002	0.91
*NIV use*	6.8	2	3.38	0.62	0.01	0.54
*Gastrostomy use*	3.9	2	1.94	0.35	0.006	0.70

^a^ANOVA uses disease sub-types of amyotrophic lateral sclerosis and progressive lateral sclerosis only.

#### Management and assessment of non-motor symptoms

Of the sub-group (*n* = 109) who responded to this question, 73% were satisfied with the frequency with which these symptoms were discussed with their clinical team, reporting that they felt able to raise concerns where needed.

Eighty per cent of all participants indicated that they believe non-motor symptoms are important and should be included in future clinical trials, either to be explored in secondary outcome measures as potential additional therapeutic benefits for candidate drugs or as areas to consider as possible side-effects.

The total number of non-motor symptoms participants personally reported did not have a significant association with the likelihood of a participant indicating ‘yes’ to the question ‘Is it important to you that trials for new drug treatments also consider symptoms like these as well?’, (odds ratio = 1.03, 95% confidence interval = 0.82–1.28, *P* = 0.79). This suggests that the participants’ personal experience of non-motor symptoms was not associated with their view that these symptoms are important to include in trial design.

The potential side-effects most frequently raised as concerning by respondents were concerns that a prospective new treatment would cause, or worsen, nausea/vomiting, gastrointestinal issues or fatigue. Ten per cent of respondents stated that any side-effects would inform their decision-making process, and 10% reported that no side-effects would prevent them from taking a drug.

### Assessment methods

The most acceptable method of data collection to participants was completing questionnaires themselves remotely, with 91% (*n* = 108) reporting this as acceptable. This was followed by completing questionnaires with the support of the research team, with 71% (*n* = 83) reporting as acceptable or using wearable devices to provide data, 68% (*n* = 79). After these methods, participants indicated a lesser preference for data collection using specialized websites or entering their data into apps. The full exploration of assessment method preference is reported in Table 7.

**Table 7:** Assessment methods ranked by preference

Assessment method	*N*	Acceptable *n* (%)	Not sure *n* (%)	Prefer not to *n* (%)
Completing questionnaires, at home, about my symptoms	119	108 (91)	4 (3)	7 (6)
Completing questionnaires in clinic, with the help of a researcher or clinician, about my symptoms	117	83 (71)	12 (10)	22 (19)
Wearing a device on my body which senses movement (similar to a FitBit wristwatch)	116	79 (68)	20 (17)	17 (15)
Type my data into a specialized website	115	66 (57)	19 (17)	30 (26)
Putting data about my symptoms into an app on my smartphone	116	49 (42)	22 (19)	45 (39)

## Discussion

### Findings summary

This study presents the results from a structured questionnaire-based study of people living with motor neuron disease in Scotland and identifies that 99% report experiencing a non-motor symptom, with 72% reporting five or more non-motor symptoms. The high prevalence and frequency of these symptoms are consistently reported in systematic reviews of prior literature,^3^ symptom-focused research^41^ and interventional studies.^12^ This study used a questionnaire-based methodology to collate feedback directly from people with motor neuron disease on their experience with a broad list of potential non-motor symptoms and suggests that these symptoms are more widely experienced and more frequently co-occur than previously suggested in symptom-specific studies.^10,15^

Pain and fatigue were the symptoms participants reported to be the most frequently occurring and most impactful on daily life, aligning with previous research findings on the multi-factorial nature and added burden of pain and fatigue for people living with motor neuron disease.^6,42,43^ In particular, the finding that pain is a frequently occurring and impactful symptom of motor neuron disease echoes findings presented by the Trajectories of Outcomes in Neurological Conditions group, who in a larger sample of 636 found 69% individuals reported pain and significantly reduced a person’s quality of life.^21^ The prevalence of non-motor symptoms also varied with the clinical characteristics of participants, with those who were long survivors and those with worse physical function (as indicated by lower ALS-FRS(R) scores) more likely to report more non-motor symptoms.

To our knowledge, this is the first study to directly ask participants about their current experience of the evaluation of non-motor symptoms in clinical care and towards the inclusion of non-motor symptoms in clinical trials.

Although symptom-focused trials in motor neuron disease are beneficial to identify treatments, our recent systematic review suggests that the additional potential benefits or side-effects of candidate drugs in motor-focused trials are under-evaluated.^29,30^ The findings from the current study indicate that people affected by motor neuron disease are also supportive of exploring the holistic impact of motor neuron disease treatment options when choosing outcome measures, irrespective of their personal experience of such symptoms.

The vast majority of people with motor neuron disease supported the consideration of non-motor symptoms when evaluating new drug treatments, aligning with pre-existing expert guidelines,^25^ which encourage a more holistic perspective of the impact, management and treatment of motor neuron disease. By focusing on the participant perspective, findings from this study inform of the broad range of symptoms that can be experienced in addition to, or as a result of, motor degeneration in motor neuron disease.

### Non-motor symptoms reported

Understanding the frequency and impact of non-motor symptoms experienced by people with motor neuron disease continues to challenge the narrative of motor neuron disease as a primarily motor disorder. A greater understanding of these symptoms can provide an insight into the heterogeneity of motor neuron disease and inform decisions regarding prospective therapeutic targets. Evaluating the importance of these symptoms to people with motor neuron disease enables us to better understand the holistic impact of motor neuron disease on the individual and the disability and impact on quality of life that occur as a result of motor degeneration.

An example of this is the improved understanding over the past decade of cognitive and behavioural change as a common aspect of motor neuron disease progression, particularly the diagnostic overlap with frontotemporal dementia.^44^ In line with clinical care guidelines, the multi-disciplinary team now routinely screens for cognitive impairment and behavioural symptoms in people with motor neuron disease, with the opportunity to use specifically developed assessment tools such as the Edinburgh Cognitive and Behavioural ALS Screen.^45^ Understanding of the frequency of these cognitive and behavioural issues has enabled early intervention so people with motor neuron disease and their supporters can implement management strategies and adapt care plans accordingly.^46^

### Evaluation of non-motor symptoms

Integration of non-motor symptoms into the trial design is key to offering holistic evaluation of disease progression and effective treatment options. Including non-motor symptoms either as additional outcome measures, potential therapeutic targets or side-effects of investigative medicinal products is supported by the overwhelming majority of respondents and aligns with current trial design guidance.^25,26^ This study is the first to offer feedback from people affected by motor neuron disease on integrating non-motor symptoms into trial design, indicating that the majority of respondents are supportive.

Holistic assessment of non-motor symptoms is also crucial in clinical care. Providing space within the multi-disciplinary team, and time within the clinical appointment schedule, is essential to ensure people with motor neuron disease feel able to discuss their changing health. Non-motor symptoms may not be reported by people with motor neuron disease, so the clinician must lead discussions in this area, prompting their patient to consider symptoms beyond their motor dysfunction and explain the various ways motor neuron disease can present.^23^

Awareness that these symptoms may also be more common in certain groups of people with motor neuron disease may also help shape clinical discussions and offer areas of focus for future research. The current study and previous research indicate that non-motor symptoms increase as the disease progresses and physical function worsens,^5,16^ with more symptoms reported by long survivors.

In designing effective research and delivering efficient clinical care, the way in which all symptoms of motor neuron disease are assessed must be a balance between patient preference and data quality. Respondents indicated a clear preference for remote data collection through at-home questionnaires, reducing the burden of travelling to appointments and enabling people with motor neuron disease to respond at their own pace. A preference for remote assessment has been indicated in larger surveys considering self-monitoring and device use as remote options for clinical care and trial delivery.^47^

### Strengths and limitations

A key strength of this study is the relatively large population-based sample size for a condition classified as a rare disorder based on prevalence data.^36,48^ A total of 120 people with motor neuron disease completed the study questionnaires, with demographics and clinical features generalizable to the wider motor neuron disease population. A secondary strength is the focus on exploring the participant perspective using prospective data, in addition to a pre-defined list of potential non-motor symptoms participants were encouraged to consider how they were affected by motor neuron disease and list symptoms that were impactful to them.

A limitation of this general area of research is the difficulty of defining non-motor symptoms, and the subjectivity of how these symptoms can affect, and be reported by, people with motor neuron disease. Different people may report the presence and impact of these symptoms differently, and this impacts their measurement. Quantification of non-motor symptoms as present versus absent, to enable comparison across participants, may be reductionist. Complex concepts such as mood and fatigue are difficult to define and often overlap, and results must be interpreted cautiously with this in mind. Some of the included symptoms may be more complex and multi-dimensional than others, affecting how participants acknowledge and report their presence.

The availability of disease-specific measurement tools is limited,^29^ and current studies use assessments designed for other neurological conditions adapted for motor neuron disease.^4^ However, these are often reliant on self-reporting of symptoms or clinical judgement, which may be flawed or subjective in their interpretation. An additional limitation of this study is a reliance on self-reporting of symptoms, which may lead to an over (or under) estimation of symptom prevalence and impact. Self-reporting also raises the issue of insight, particularly in relation to cognitive and behavioural changes, that the person affected by motor neuron disease may not report due to lack of awareness of these issues, and as a result, these may be under-represented in the current study.

### Future research

Future research in this area may focus on the benefits of assessing non-motor symptoms within current multi-disciplinary care teams and evaluating the suitability of new symptom-focused interventions as potential avenues to improve the quality of life for those currently living with motor neuron disease. This may be a particularly impactful area in individuals in the earlier stages of the condition, and exploring the pattern of non-motor symptoms in those individuals with a recent diagnosis offers the opportunity to evaluate the benefit of early intervention and the impact of symptoms on care planning decisions.

In addition, studies could explore how these non-motor symptoms can be explored as additional outcome measures in clinical trials. In previous reviews,^29,30^ we have reported that non-motor symptoms have been under-evaluated in trial design and the current study provides evidence of support from prospective participants to include additional assessments. More randomized controlled trials focusing on the impact of interventions and medication regimens on non-motor symptoms in motor neuron disease will enable clinicians to make informed, evidenced-based, management decisions that consider the holistic impact of the condition. Considering the effectiveness of interventions for the non-motor symptoms is a key direction to explore, and the findings will have implications for clinical care.

## Conclusion

Ninety-nine per cent of people with motor neuron disease experience at least one non-motor symptom and 72% report five or more; these symptoms affect the vast majority of people with motor neuron disease and often co-occur. The most frequently occurring symptoms are pain and fatigue, reported by 76% of participants respectively, and symptoms that have the most significant impact, where reported, are pain and problematic saliva. Evaluation of the impact of these symptoms on function and quality of life is necessary to understand, manage and ultimately treat the individual. The frequency of reporting non-motor symptoms increases in long survivors and for those with worsened physical function.

The key novel aspect of this study was the exploration of the attitudes of people with motor neuron disease towards including the evaluation of non-motor symptoms in clinical trials. Findings indicated that people with motor neuron disease, prospective participants in these trials, were supportive of including non-motor assessments in trials, in line with clinical^19^ and trial^25^ guidance encouraging a broader conceptualization of treatment.

## Supplementary material

Supplementary material is available at *Brain Communications* online.

## Supplementary Material

fcad336_Supplementary_Data

## Data Availability

Data are available as a Supplementary material to this manuscript, with identifying information removed.

## Appendices

### Appendix 1: Study questionnaires

#### Non-notor symptoms in motor neuron disease

Motor neuron disease affects everyone differently. Below is a list of symptoms that may affect some people with motor neuron disease with different levels of seriousness.

**Table:**

		Have you experienced this issue? *If no, please move on to the next symptom.*	How frequently did this happen in the last 2 weeks?	Is this a significant problem for you?	Can you describe how this affects you?
Sleep	Problems such as struggling to get to, or stay, asleep, sleeping too much and snoring when asleep	( ) Yes ( ) No	( ) A few days ( ) Most days ( ) Every day	( ) Yes ( ) No ( ) Unsure	
Saliva	Problems with thick saliva or drooling	( ) Yes ( ) No	( ) A few days ( ) Most days ( ) Every day	( ) Yes ( ) No ( ) Unsure	
Pain	Cramps	( ) Yes ( ) No	( ) A few days ( ) Most days ( ) Every day	( ) Yes ( ) No ( ) Unsure	
Low mood	Feeling sad or ‘blue’	( ) Yes ( ) No	( ) A few days ( ) Most days ( ) Every day	( ) Yes ( ) No ( ) Unsure	
Anxiety	Feeling anxious, worried or restless	( ) Yes ( ) No	( ) A few days ( ) Most days ( ) Every day	( ) Yes ( ) No ( ) Unsure	
Fatigue	Feeling tired and exhausted even when you have had plenty of rest or sleep	( ) Yes ( ) No	( ) A few days ( ) Most days ( ) Every day	( ) Yes ( ) No ( ) Unsure	
Cognition	Problems such as feeling your thinking is slowed, struggling to find the right word or difficulties planning and completing tasks	( ) Yes ( ) No	( ) A few days ( ) Most days ( ) Every day	( ) Yes ( ) No ( ) Unsure	
Behaviour	Problems such as crying or laughing when you do not mean to, struggling to find motivation, having difficulty understanding how other people are feeling or a change in your food preference	( ) Yes ( ) No	( ) A few days ( ) Most days ( ) Every day	( ) Yes ( ) No ( ) Unsure	
Sexuality	Change in sexual drive, ability to become physically aroused, reaching orgasm or physical capacity to have sex	( ) Yes ( ) No	( ) A few days ( ) Most days ( ) Every day	( ) Yes ( ) No ( ) Unsure	
Gastro intestinal	Issues such as constipation, nausea, vomiting, acid reflux or flatulence	( ) Yes ( ) No	( ) A few days ( ) Most days ( ) Every day	( ) Yes ( ) No ( ) Unsure	

Do your clinical team ask you about these symptoms when you go to appointments? Would you like them to ask you about any of these symptoms more often?

__________________________________________________________________________________________________________ __________________________________________________________________________________________________________

Is it important to you that trials for new drug treatments also consider symptoms like these as well?

__________________________________________________________________________________________________________ __________________________________________________________________________________________________________

What types of side-effects would concern you most about new treatments being investigated in a drug trial? If a drug being investigated in a clinical trial led to side-effects, which side-effects would stop you from wanting to take a new drug?

__________________________________________________________________________________________________________ __________________________________________________________________________________________________________

#### Participant-reported symptoms

Please ‘list up to five symptoms’ that you experience, which affect your life the most. If you cannot think of this many, please leave lines blank. You can use symptoms already mentioned in the questionnaire or new ones of your own.

Please indicate how often this symptom occurs for you, and how much of a problem it is for you.

**Table:**

No.	Symptom	How often did it affect you in the past 2 weeks?	How much of a problem is it?
1	__________________________________________	[ ] Over half the days [ ] Under half the days	[ ] This is a significant problem for me. [ ] This bothers me from time to time. [ ] This happens but does not affect me much.
2	__________________________________________	[ ] Over half the days [ ] Under half the days	[ ] This is a significant problem for me. [ ] This bothers me from time to time. [ ] This happens but does not affect me much.
3	__________________________________________	[ ] Over half the days [ ] Under half the days	[ ] This is a significant problem for me. [ ] This bothers me from time to time. [ ] This happens but does not affect me much.
4	__________________________________________	[ ] Over half the days [ ] Under half the days	[ ] This is a significant problem for me. [ ] This bothers me from time to time. [ ] This happens but does not affect me much.
5	__________________________________________	[ ] Over half the days [ ] Under half the days	[ ] This is a significant problem for me. [ ] This bothers me from time to time. [ ] This happens but does not affect me much.

#### Assessment of symptoms

If you were being asked about your motor neuron disease symptoms in clinical or research appointments, there are many different ways that we can collect this information.

We would like to know your preferences on how to collect information on motor neuron disease symptoms.

Below is a list of ways that we can collect information, please use the tick boxes to indicate if you would find these options acceptable or not acceptable.

**Table:**

Number	Option	Acceptable	Not sure	Prefer not to
1	Completing questionnaires, at home, about my symptoms			
2	Completing questionnaires in clinic, with the help of a researcher or clinician, about my symptoms			
3	Putting data about my symptoms into an app on my smartphone			
4	Type my data into a specialized website			
5	Wearing A Device On My Body Which Senses Movement (Similar To A Fitbit Wristwatch)			

### Appendix 2: ALSFRS(R)

#### Speech

**Table:**

*How is your speech?*		
*Please tick*		
1.	My speech is normal; there has been no change since my diagnosis.	
2.	There is a change in my speech that people (either yourself or others) have noticed.	
3.	People can understand me when I speak but I have to repeat myself often (around a quarter of the time, or more).	
4.	I can speak but I also need to use technology or writing to be understood.	
5.	I can no longer speak.	

#### Saliva

**Table:**

*How is your saliva?*		
*Please tick*		
1.	I do not have any excessive saliva (please still tick this option if you have a dry mouth).	
2.	I feel I have excessive saliva and there may be some drooling at night, but I do not usually have to mop up saliva with a tissue.	
3.	I need a use a tissue to mop up excessive saliva, but not often (less than a quarter of the time).	
4.	I experience drooling and have to use a tissue to mop up excessive saliva often, but not all the time.	
5.	I need to use a tissue, or suction device, to mop up excessive saliva all of the time.	

#### Swallowing

**Table:**

*How is your swallowing?*		
*Please tick*		
1.	I have no problem eating food and having drinks the same as I did before my motor neuron disease diagnosis.	
2.	I have some issues with food sticking in my throat or coughing/choking when I eat. Sometimes I have to cut food up small, but I never have to mash or liquidize food.	
3.	I need to have food mashed or liquidized, or my drinks need thickeners in. I avoid tougher and drier foods.	
4.	I struggle to eat food and I need to have gastrostomy to add to my calories intake (please tick yes if you need gastrostomy, regardless of if you have one or not).	
5.	I only take in calories through gastrostomy or other supported feeding.	

#### Handwriting

**Table:**

*Thinking about your dominant hand, are you able to hold a pen?* *Please tell us about your handwriting.*		
1.	My handwriting is normal there has been no change.	
2.	My handwriting is slower and sloppier but all words are legible, or sometimes I use writing aids or specialized pen grips.	
3.	Not all of my words are legible when I write.	
4.	I can grip a pen, but my words are not legible.	
5.	I am unable to grip a pen.	

*A. Cutting food and handling utensils—without gastrostomy*

**Table:**

*Please complete this section if you do not use gastrostomy as your only source of calories. If you only use gastrostomy, please move on to question 5B below.* *How are you with cutting food or handling cutlery?*		
1.	There has been no change in my ability to cut up my food and I have not changed the utensils I use to eat.	
2.	I am a little slow and clumsy, but I do not need help from others.	
3.	Occasionally I need help from others to eat, but usually I can do it alone.	
4.	I need someone else to cut up my food, but I am able to eat independently.	
5.	I need some to cut up my food and help me with eating.	

*B. Cutting food and handling utensils—with gastrostomy*

**Table:**

*If you do not have gastrostomy, or still have some food, please complete 5A instead.* *How are you with handling the gastrostomy fastenings and fixtures?*		
1.	I have no difficulty with setting up the fastenings and fixtures.	
2.	I am a little slower and clumsy but I can do it myself.	
3.	I need some help from others with the closures and fasteners.	
4.	I need someone else to do most of the setting up.	
5.	I am unable to do the task alone, someone else has to do it for me.	

#### Dressing and hygiene

**Table:**

*How are you with dressing or washing?*		
1.	There has been no change since my motor neuron disease began.	
2.	I am a little slower but I do not need other people or supportive devices (e.g. button hook) to help me.	
3.	I need some assistance at some stages from other people, or supportive devices, when I am getting dressed or washing, but I can do most of it myself.	
4.	I need assistance when I am getting dressed or washing, but I can do some parts myself.	
5.	I cannot dress or bathe myself and need support from others.	

#### Turning in bed and adjusting bed clothes

**Table:**

*Can you turn in bed and adjust the bed clothes? (pillow/blankets)*		
1.	I can do this as normal.	
2.	I am a little slower and clumsy but I can do this without help.	
3.	I can turn, or adjust the bed sheets, alone but with great difficulty.	
4.	I can begin to turn or adjust the bed sheets but I need someone to help me.	
5.	I cannot turn or adjust the bedsheets.	

#### Walking

**Table:**

*How is your walking?*		
1.	I can walk as normal.	
2.	I am have some issues with walking slowly, tripping or balance but I do not need help from others or a walking aid.	
3.	I can walk with help from others or a walking aid.	
4.	I can stand up and weight bear.	
5.	I can no longer walk.	

#### Climbing up stairs

**Table:**

*Are you able to climb up stairs? Please only think about going up, not down.*		
1.	I have no problems going up stairs.	
2.	I am a little slower but I do not feel unsteady or need to rest between steps.	
3.	I need to rest between steps and I feel unsteady.	
4.	I need support from another person or a handrail to be able to climb stairs.	
5.	I cannot climb stairs.	

#### Breathlessness

**Table:**

*Do you become breathless? If you are using non-invasive ventilation please choose the final option.*		
1.	No I do not become breathless.	
2.	I get breathless when I am walking (walking at a good speed on a flat route).	
3.	I get breathless when I am eating, bathing or dressing.	
4.	I get breathless when I am sitting or lying down.	
5.	I have significant difficulty breathing and I either currently use, or am considering using, a ventilation system.	

#### Lying down

**Table:**

*Can you sleep lying down flat or do you need to be propped up?*		
1.	I do not need to be propped up.	
2.	I have some difficulty sleeping at night due to shortness of breath, but I do not routinely use more than two pillows.	
3.	I need more than two pillows to sleep, or I need to be angled at 45° or more.	
4.	I can only sleep sitting up in bed or a chair.	
5.	I can only sleep with non-invasive mechanical ventilation on for most, or all, of the night.	

#### Respiratory insufficiency

**Table:**

*Do you use non-invasive ventilation?*		
1.	I do not use.	
2.	I use ventilation occasionally.	
3.	I use ventilation during the night only.	
4.	I use ventilation during the day and night.	
5.	I have permanent invasive ventilation by intubation or tracheostomy.	

### Appendix 3: CARE of motor neuron disease clinical data

In addition to data collection through questionnaires, this project involved a data request to CARE of motor neuron disease for clinical information on participants.

**Table:**

Area	Data
Demographics	Age at diagnosis Sex Health board Date of diagnosis Date of death
Phenotype	Motor neuron disease sub-type Site of onset Areas currently affected
Interventions use	Riluzole Non-invasive ventilation Gastrointestinal feeding tube insertion
Cognition and behaviour	Date of cognitive assessment Edinburgh Cognition Assessment Screen score for cognition and behaviour^46^
Speech	Referral to Speech and Language Therapist Sialorrhoea Problematic saliva
Physical symptoms	Additional Amyotrophic Lateral Sclerosis Functional Rate Scale—Revised (ALSFRS(R)-r) data where required due to self-reported data missing^17^ Additional symptoms and date of onset
Research Participation	MND-SMART enrolment Other research projects consented to

## References

[1] Black HA, Leighton DJ, Cleary EM, et al Genetic epidemiology of motor neuron disease-associated variants in the Scottish population. Neurobiol Aging. 2017;51:178.e11–178.e20. 10.1016/j.neurobiolaging.2016.12.013 PMC5302213 28089114

[2] Benbrika S, Desgranges B, Eustache F, Viader F. Cognitive, emotional and psychological manifestations in amyotrophic lateral sclerosis at baseline and overtime: A review. Front Neurosci. 2019;13:951. 31551700 10.3389/fnins.2019.00951 PMC6746914

[3] Fang T, Jozsa F, Al-Chalabi A. Nonmotor symptoms in amyotrophic lateral sclerosis: A systematic review. Int Rev Neurobiol. 2017;134:1409–1441. 28805578 10.1016/bs.irn.2017.04.009

[4] Shojaie A, Rota S, Al Khleifat A, Ray Chaudhuri K, Al-Chalabi A. Non-motor symptoms in amyotrophic lateral sclerosis: Lessons from Parkinson’s disease. Amyotroph Lateral Scler Frontotemporal Degener. 2023. 10.1080/21678421.2023.2220748 37349906

[5] Günther R, Richter N, Sauerbier A, et al Non-motor symptoms in patients suffering from motor neuron diseases. Front Neurol. 2016;7:117. 27504105 10.3389/fneur.2016.00117 PMC4958907

[6] Chiò A, Canosa A, Gallo S, et al Pain in amyotrophic lateral sclerosis: A population-based controlled study. Eur J Neurol. 2012;19(4):551–555. 21972798 10.1111/j.1468-1331.2011.03540.x

[7] Nash Y, Sitty M. Non-motor symptoms of amyotrophic lateral sclerosis: A multi-faceted disorder. J Neuromuscular Dis. 2021;8(4):699–713. 10.3233/JND-210632 34024773

[8] Christidi F, Karavasilis E, Rentzos M, et al Clinical and radiological markers of extra-motor deficits in amyotrophic lateral sclerosis. Front Neurol. 2018;9:1005. 30524366 10.3389/fneur.2018.01005 PMC6262087

[9] Chenji S, Ishaque A, Mah D, et al Neuroanatomical associations of the Edinburgh Cognitive and Behavioural ALS screen (ECAS). Brain Imaging Behav. 2021;15(3):1641–1654. 33155172 10.1007/s11682-020-00359-7

[10] Raheja D, Stephens HE, Lehman E, et al Patient-reported problematic symptoms in an ALS treatment trial. Amyotroph Lateral Scler Frontotemporal Degener. 2016;17(3–4):198–205. 26824413 10.3109/21678421.2015.1131831 PMC4979603

[11] The ALS CNTF Treatment Study (ACTS) Phase I-II Study Group . The amyotrophic lateral sclerosis functional rating scale. Assessment of activities of daily living in patients with amyotrophic lateral sclerosis. Arch Neurol. 1996;53:141–147. 8639063

[12] McClelland S III, Bethoux FA, Boulis NM, et al Intrathecal baclofen for spasticity-related pain in amyotrophic lateral sclerosis: Efficacy and factors associated with pain relief. Muscle Nerve. 2008;37(3):396–398. 17894358 10.1002/mus.20900

[13] Katzberg HD, Benatar M. Enteral tube feeding for amyotrophic lateral sclerosis/motor neuron disease. Cochrane Database Syst Rev. 2011;2011(1):CD004030. 21249659 10.1002/14651858.CD004030.pub3 PMC7163276

[14] Opie-Martin S, Ossher L, Bredin A, et al Motor neuron disease register for England, Wales and Northern Ireland—An analysis of incidence in England. Amyotroph Lateral Scler Frontotemporal Degener. 2021;22(1–2):86–93. 32940088 10.1080/21678421.2020.1812661

[15] McHutchison CA, Leighton DJ, McIntosh A, et al Relationship between neuropsychiatric disorders and cognitive and behavioural change in MND. J Neurol Neurosurg Psychiatry. 2020;91(3):245–253. 31871139 10.1136/jnnp-2019-321737

[16] Devenney EM, McErlean K, Tse NY, et al Factors that influence non-motor impairment across the ALS-FTD spectrum: Impact of phenotype, sex, age, onset and disease stage. Front Neurol. 2021;12:743688. 34899567 10.3389/fneur.2021.743688 PMC8656429

[17] Young CA, Ealing J, McDermott C, et al The relationships between symptoms, disability, perceived health and quality of life in amyotrophic lateral sclerosis/motor neuron disease. Amyotroph Lateral Scler Frontotemporal Degener. 2019;20(5–6):317–327. 31116037 10.1080/21678421.2019.1615951

[18] van Groenestijn AC, Kruitwagen-van Reenen ET, Visser-Meily JMA, van den Berg LH, Schröder CD. Associations between psychological factors and health-related quality of life and global quality of life in patients with ALS: A systematic review. Health Qual Life Outcomes. 2016;14(1):107. 27439463 10.1186/s12955-016-0507-6 PMC4955215

[19] National Institute for Health and Care Excellence, C.G.C.U., Motor Neurone Disease: Assessment and Management. 2016. Accessed 2021. https://www.nice.org.uk/guidance/ng42 26962594

[20] Chiò A, Canosa A, Calvo A, et al Developments in the assessment of non-motor disease progression in amyotrophic lateral sclerosis. Expert Rev Neurother. 2021;21(12):1419–1440. 34554894 10.1080/14737175.2021.1984883

[21] Edge R, Mills R, Tennant A, et al Do pain, anxiety and depression influence quality of life for people with amyotrophic lateral sclerosis/motor neuron disease? A national study reconciling previous conflicting literature. J Neurol. 2020;267:607–615. 31696295 10.1007/s00415-019-09615-3 PMC7035222

[22] Ando H, Cousins R, Young CA. Flexibility to manage and enhance quality of life among people with motor neurone disease. Disabil Rehabil. 2013;44(12):2752–2762. 10.1080/09638288.2020.1846797 33226867

[23] Chaudhuri KR, Healy DG, Schapira AH. Non-motor symptoms of Parkinson's disease: Diagnosis and management. Lancet Neurol. 2006;5(3):235–245. 16488379 10.1016/S1474-4422(06)70373-8

[24] Ng L, Khan F, Young CA, Galea M. Symptomatic treatments for amyotrophic lateral sclerosis/motor neuron disease. Cochrane Database Syst Rev. 2017;1(1):CD011776. 28072907 10.1002/14651858.CD011776.pub2 PMC6469543

[25] Van Den Berg LH, Sorenson E, Gronseth G, et al Revised Airlie House consensus guidelines for design and implementation of ALS clinical trials. Neurology. 2019;92(14):e1610–e1623. 30850440 10.1212/WNL.0000000000007242 PMC6448453

[26] European Medicines Agency . Clinical investigation of medicinal products for the treatment of amyotrophic lateral sclerosis. EMA/531686/2015, Corr.1, 2015. https://www.ema.europa.eu/en/documents/scientific-guideline/guideline-clinical-investigation-medicinal-products-treatment-amyotrophic-lateral-sclerosis_en.pdf

[27] van Eijk RP, Kliest T, van den Berg LH. Current trends in the clinical trial landscape for amyotrophic lateral sclerosis. Curr Opin Neurol. 2020;33(5):655–661. 32796282 10.1097/WCO.0000000000000861

[28] Mahoney CJ, Ahmed RM, Huynh W, et al Pathophysiology and treatment of non-motor dysfunction in amyotrophic lateral sclerosis. CNS Drugs. 2021;35(5):483–505. 33993457 10.1007/s40263-021-00820-1

[29] Beswick E, Forbes D, Hassan Z, et al A systematic review of non-motor symptom evaluation in clinical trials for amyotrophic lateral sclerosis. J Neurol. 2021;268(12):4510–4521. 34120226 10.1007/s00415-021-10651-1 PMC8738361

[30] Beswick E, Park E, Wong C, et al A systematic review of neuropsychiatric and cognitive assessments used in clinical trials for amyotrophic lateral sclerosis. J Neurol. 2020;268(12):4510–4521. 32910255 10.1007/s00415-020-10203-z PMC8563523

[31] Leighton D, Newton J, Colville S, et al Clinical audit research and evaluation of motor neuron disease (CARE-MND): A national electronic platform for prospective, longitudinal monitoring of MND in Scotland. Amyotroph Lateral Scler Frontotemporal Degener. 2019;20(3–4):242–250. 30889975 10.1080/21678421.2019.1582673

[32] Cedarbaum JM, Stambler N, Malta E, et al The ALSFRS-R: A revised ALS functional rating scale that incorporates assessments of respiratory function. BDNF ALS Study Group (Phase III). J Neurol Sci. 1999;169(1–2):13–21. 10540002 10.1016/s0022-510x(99)00210-5

[33] Bakker LA, Schröder CD, Tan HH, et al Development and assessment of the inter-rater and intra-rater reproducibility of a self-administration version of the ALSFRS-*R*. J Neurol Neurosurg Psychiatry. 2020;91(1):75–81. 31558653 10.1136/jnnp-2019-321138

[34] Meyer T, Spittel S, Grehl T, et al Remote digital assessment of amyotrophic lateral sclerosis functional rating scale–a multicenter observational study. Amyotroph Lateral Scler Frontotemporal Degener. 2022;24(3–4):175–184. 35912984 10.1080/21678421.2022.2104649

[35] Chiò A, Moglia C, Canosa A, et al ALS phenotype is influenced by age, sex, and genetics: A population-based study. Neurology. 2020;94(8):e802–e810. 31907290 10.1212/WNL.0000000000008869

[36] Leighton DJ, Newton J, Stephenson LJ, et al Changing epidemiology of motor neurone disease in Scotland. J Neurol. 2019;266(4):817–825. 30805795 10.1007/s00415-019-09190-7

[37] Raymond J, Oskarsson B, Mehta P, Horton K. Clinical characteristics of a large cohort of US participants enrolled in the National Amyotrophic Lateral Sclerosis (ALS) Registry, 2010–2015. Amyotroph Lateral Scler Frontotemporal Degener. 2019;20(5–6):413–420. 31131638 10.1080/21678421.2019.1612435 PMC6946020

[38] O'Neill CL, Williams TL, Peel ET, et al Non-invasive ventilation in motor neuron disease: An update of current UK practice. J Neurol Neurosurg Psychiatry. 2012;83(4):371–376. 21849339 10.1136/jnnp-2011-300480

[39] Tartaglia MC, Rowe A, Findlater K, et al Differentiation between primary lateral sclerosis and amyotrophic lateral sclerosis: Examination of symptoms and signs at disease onset and during follow-up. Arch Neurol. 2007;64(2):232–236. 17296839 10.1001/archneur.64.2.232

[40] Leighton DJ, Ansari M, Newton J, et al Genotype–phenotype characterisation of long survivors with motor neuron disease in Scotland. J Neurol. 2023;270(3):1702–1712. 36515702 10.1007/s00415-022-11505-0 PMC9971124

[41] Ferentinos P, Paparrigopoulos T, Rentzos M, et al Prevalence of major depression in ALS: Comparison of a semi-structured interview and four self-report measures. Amyotroph Lateral Scler. 2011;12(4):297–302. 21428731 10.3109/17482968.2011.556744

[42] Rivera I, Ajroud-Driss S, Casey P, et al Prevalence and characteristics of pain in early and late stages of ALS. Amyotroph Lateral Scler Frontotemporal Degener. 2013;14(5–6):369–372. 23286754 10.3109/21678421.2012.751614

[43] Wallace VC, Ellis CM, Burman R, et al The evaluation of pain in amyotrophic lateral sclerosis: A case controlled observational study. Amyotroph Lateral Scler Frontotemporal Degener. 2014;15(7–8):520–527. 25204842 10.3109/21678421.2014.951944

[44] Burrell JR, Halliday GM, Kril JJ, et al The frontotemporal dementia-motor neuron disease continuum. Lancet. 2016;388(10047):919–931. 26987909 10.1016/S0140-6736(16)00737-6

[45] Niven E, Newton J, Foley J, et al Validation of the Edinburgh Cognitive and Behavioural Amyotrophic Lateral Sclerosis Screen (ECAS): A cognitive tool for motor disorders. Amyotroph Lateral Scler Frontotemporal Degener. 2015;16(3–4):172–179. 25967542 10.3109/21678421.2015.1030430

[46] Hodgins F, Mulhern S, Abrahams S. The clinical impact of the Edinburgh Cognitive and Behavioural ALS Screen (ECAS) and neuropsychological intervention in routine ALS care. Amyotroph Lateral Scler Frontotemporal Degener. 2019;21(1–2):92–99. 31612737 10.1080/21678421.2019.1674874

[47] Helleman J, Johnson B, Holdom C, et al Patient perspectives on digital healthcare technology in care and clinical trials for motor neuron disease: An international survey. J Neurol. 2022;269(11):6003–6013. 35849154 10.1007/s00415-022-11273-x PMC9294855

[48] Logroscino G, Traynor BJ, Hardiman O, et al Descriptive epidemiology of amyotrophic lateral sclerosis: New evidence and unsolved issues. J Neurol Neurosurg Psychiatry. 2008;79(1):6–11. 18079297 10.1136/jnnp.2006.104828

[49] Boentert M . Sleep disturbances in patients with amyotrophic lateral sclerosis: current perspectives. Nat Sci Sleep. 2019;11:97. 31496852 10.2147/NSS.S183504 PMC6701267

[50] Ohayon MM , Priest RG, Guilleminault C, Caulet M. The prevalence of depressive disorders in the United Kingdom. Biol Psychiatry. 1999;45(3):300–307. 10023506 10.1016/s0006-3223(98)00011-0

[51] Wittchen H-U, Jacobi F, Rehm J, et al The size and burden of mental disorders and other disorders of the brain in Europe. Eur Neuropsychopharmacol. 2010;21(9):655–679. 10.1016/j.euroneuro.2011.07.018 21896369

[52] Chio A, Logroscino G, Hardiman O, et al Prognostic factors in ALS: a critical review. Amyotroph Lateral Scler. 2009;10(5-6):310–323. 19922118 10.3109/17482960802566824 PMC3515205

[53] Chiò A, Vignola A, Mastro E, et al Neurobehavioral symptoms in ALS are negatively related to caregivers’ burden and quality of life. Europ Jou Neurol. 2010;17(10):1298–1303. 10.1111/j.1468-1331.2010.03016.x 20402747

[54] Gibbons ZC, Richardson A, Neary D, Snowden JS. Behaviour in amyotrophic lateral sclerosis. Amyotrop Lateral Scler. 2008;9(2):67–74. 10.1080/17482960701642437 18427998

[55] Radakovic R, Stephenson L, Colville S, Swingler R, Chandran S, Abrahams S. Multidimensional apathy in ALS: validation of the Dimensional Apathy Scale. Jou Neurol. Neurosurg Psychiatry. 2016;87(6):663–669. 10.1136/jnnp-2015-310772 26203157

[56] Pearson I, Glasmacher SA, Newton J, et al The prevalence and management of saliva problems in motor neuron disease: A 4-year analysis of the Scottish MND Register. Neurodegener Dis. 2021. (In Press). 10.1159/000514615 PMC7610776 33902047

[57] Garuti G, Rao F, Ribuffo V, Sansone VA. Sialorrhea in patients with ALS: current treatment options. Degener Neurol Neuromuscul Dis. 2019;9:19. 31118868 10.2147/DNND.S168353 PMC6498144

[58] Toepfer M, Folwaczny C, Klauser A, Riepl RL, Müller-Felber W, Pongratz D. Gastrointestinal dysfunction in amyotrophic lateral sclerosis. Amyotrop Lateral Scler Other Motor Neuron Disord. 2000;1(1):15–19. 10.1080/146608299300079484 12365061

[59] Ahmed RM, Ke YD, Vucic S, et al Physiological changes in neurodegeneration-mechanistic insights and clinical utility. Nat Rev Neurol. 2018;14(5):259. 29569624 10.1038/nrneurol.2018.23

[60] Wasner M, Bold U, Vollmer TC, Borasio GD. Sexuality in patients with amyotrophic lateral sclerosis and their partners. Jou Neurol. 2004;251(4):445–448. 10.1007/s00415-004-0351-1 15083290

[61] Poletti B , Carelli L, Solca F, *et al*. Sexuality and intimacy in ALS: systematic literature review and future perspectives. Jou Neurol Neurosurg Psychiatry. 2019;90(6):712–719. 10.1136/jnnp-2018-319684 30538137

[62] The Amyotrophic Lateral Sclerosis Functional Rating Scale . Assessment of activities of daily living in patients with amyotrophic lateral sclerosis. The ALS CNTF treatment study (ACTS) phase I‐II Study Group. Arch Neurol. 1996;53:141. 8639063

