# Supplementary material for: Non-motor symptoms in motor neuron disease: prevalence, assessment and impact
Source: Brain Commun. 2023 Dec 7;6(1):fcad336. doi: 10.1093/braincomms/fcad336 (PMC10754319; doi:10.1093/braincomms/fcad336)
Supplement: fcad336_Supplementary_Data [file fcad336_supplementary_data.zip › Supplementary Table 1.docx]

## Supplementary Table 1: Participant Reported Impactful Symptoms

| **Symptom Grouping** | **Symptom Reported** | **Number of Times Symptom is Identified as Present by Participants^1^** | **Frequency of Symptom Reported as Occurring in the Past Two Weeks^2^** | | **Frequency of the Participant Indicating the Significance of the Symptom^2^** | | |
| --- | --- | --- | --- | --- | --- | --- | --- |
|  |  |  | Over Half the Days (%) | Under Half the Days (%) | This is a significant problem  for me (%) | This bothers me from  time to  time (%) | This happens but does not affect me much (%) |
| **Muscle** | *Weakness/Stiffness* | 56 | 55 (98) | 1 (2) | 43 (77) | 17 (30) | 2 (4) |
|  | *Fasciculations* | 8 | 5 (63) | 2 (3) | 3 (38) | 2 (25) | 2 (25) |
| **Mobility** | *Loss of Limb Function* | 45 | 42 (93) | 1 (2) | 32 (71) | 8 (18) | 1 (2) |
|  | *Walking* | 36 | 35 (97) | - | 33 (92) | 1 (3) | 1 (3) |
|  | *Balance/Falls* | 16 | 15 (94) | 1 (6) | 14 (88) | 2 (13) | - |
| **Oral** | *Speech* | 34 | 30 (88) | 2 (6) | 24 (71) | 9 (26) | 1 (3) |
|  | *Swallow/Choking* | 24 | 22 (92) | 2 (8) | 17 (77) | 7 (29) | - |
|  | *Breathing* | 23 | 22 (96) | 1 (4) | 20 (87) | 3 (13) | - |
|  | *Saliva* | 15 | 13 (87) | 1 (7) | 11 (73) | 3 (20) | - |
|  | *Cough* | 5 | 4 (8) | 1 (20) | 3 (60) | 2 (40) | - |
| **Tiredness** | *Fatigue* | 33 | 31 (94) | 2 (6) | 21 (64) | 11 (33) | 1 (3) |
|  | *Sleep* | 14 | 13 (93) | 1 (7) | 9 (64) | 4 (29) | 1 (7) |
| **Pain** | *Pain* | 26 | 26 (100) | - | 19 (73) | 7 (27) | - |
|  | *Cramps* | 19 | 13 (68) | 6 (32) | 11 (58) | 6 (32) | 2 (11) |
| **Quality of Life** | *Independence in Activities of Daily Living* | 17 | 17 (100) | - | 15 (88) | 1 (6) | - |
|  | *Quality of Life* | 4 | 2 (50) | 2 (50) | 3 (75) |  | 1 (25) |
| **Cognition or Behaviour** | *Behaviour Change* | 9 | 4 (45) | 5 (55) | 3 (33) | 5 (56) | 1 (11) |
|  | *Emotional Lability* | 7 | 3 (43) | 4 (57) | 3 (43) | 4 (57) | - |
|  | *Cognition* | 5 | 3 (60) | 2 (40) | 3 (60) | 2 (40) | - |
| **Gastro-intestinal** | *Constipation* | 9 | 4 (45) | 5 (55) | 3 (33) | 5 (56) | 1 (11) |
|  | *Gastrointestinal (unspecified)* | 3 | 2 (67) | 1 (33) | 2 (67) | 1 (33) | - |
|  | *Nausea* | 2 | 1 (50) | 1 (50) | 1 (50) | 1 (50) | - |
|  | *Diarrhoea* | 2 | - | 2 (100) | - | 2 (100) | - |
|  | *Acid reflux* | 1 | - | 1 (100) | - | 1 (100) | - |
| **Mood** | *Low mood* | 7 | 4 (57) | 3 (43) | 3 (43) | 4 (57) | - |
|  | *Anxiety* | 3 | 2 (67) | 1 (33) | - | 2 (67) | 1 (33) |
| **Continence** | *Incontinence* | 2 | 2 (100) | - | 2 (100) | - | - |
|  | *Nocturia* | 1 | - | - | 1 (100) | - | - |
|  | *Catheter Use* | 1 | 1 (100) | - | - | 1 (100) | - |
| 1 Note that due to missing data patterns, frequency of identification may be greater than frequency of occurrence or impact and percentages may not total 100%  2 Represented as a percentage of the number of people who reported this symptom as occurring to them | | | | | | | |
